# Mycotoxins‐contaminated wheat matrices bioconversion by *Tenebrio molitor* larvae (Coleoptera: Tenebrionidae)

**DOI:** 10.1111/1744-7917.70066

**Published:** 2025-05-13

**Authors:** Valentina Candian, Valentina Scarpino, Alessandro Bona, Rosemarie Tedeschi, Massimo Blandino

**Affiliations:** ^1^ Department of Agricultural Forest and Food Sciences (DISAFA) University of Turin Grugliasco Italy

**Keywords:** deoxynivalenol‐3‐glucoside, enniatins, exuviae, frass, moniliformin, zearalenone

## Abstract

*Tenebrio molitor* is proposed for the valorization of mycotoxins‐contaminated substrates no longer usable for other purposes. Larvae were reared on three different wheat matrices (wholegrain flour, shorts and bran) contaminated with different levels of mycotoxins. Nine diets (3 matrices × 3 contamination levels) were assessed. Larval development time, average daily gain (ADG), substrate consumption, survival rate, and the capacity of *T. molitor* to accumulate and excrete different mycotoxins, through exuviae and frass, were evaluated. Larval development time, ADG, survival rate and substrate consumption were not negatively affected by the mycotoxins, but only by the rearing matrix, depending on the nutritive value. The ability of larvae to excrete DON and its derivatives through exuviae and frass was observed. Within each matrix, DON contamination in larvae increased significantly moving from poorly to highly contaminated ones. Overall, the ratio between the modified form (deoxynivalenol‐3‐glucoside) and the native one (DON), increased from 7%, in the raw materials, to 101% in the larvae, highlighting their ability to modify this mycotoxin and accumulate it in their body. Larvae accumulated also nivalenol, zearalenone, and enniatins showing a higher concentration in larvae reared on substrates with high level of contamination, while moniliformin was never found in larvae. Overall, the levels of mycotoxins recorded in larvae was always below the current legal limits for livestock feed, thanks to their ability to excrete them through exuviae and frass. These interesting data open new scenarios on the valorization of mycotoxin‐contaminated matrices, not suitable for other livestock farming, by means of insects.

## Graphical abstract

- Larval development time, ADG, survival rate and substrate consumption were not negatively affected by the levels of mycotoxins contamination
- Larvae excreted most of the ingested DON and its derivatives through exuviae and frass
- The mycotoxin accumulation rates observed in larvae were always below the current legal limits for livestock feed

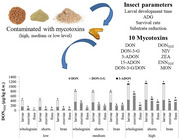

## Introduction

Mycotoxin contamination of agricultural crops is a serious threat to human and animal health, due to their acute and chronic toxicity (Rao *et al.*, 2011). FAO estimated that more than 25% of agro‐foods worldwide are contaminated by mycotoxins (FAO, 2003) and their incidence is supposed to increase in the future years. Several fungal species able to produce mycotoxin could affect different agricultural commodities, but *Fusarium* infection during the cultivation of cereal and the contamination of deoxynivalenol (DON) at grain harvest in wheat and other cereals is a widespread problem in several producing areas and global warming has been reckoned to facilitate the occurrence of mycotoxins (Medina *et al.*, 2017; Moretti *et al.*, 2019). Regulatory agencies establish guidelines and standards with maximum levels of mycotoxin residues in food. The standards established by the European Union are stricter compared to other countries (Bisconsin‐Junior *et al.*, 2023). If the concentration exceeds the limits permitted by European EC Regulation No 2023/915 and No 2024/1022 for humans, and Directive 2002/32/EC and Recommendation (EC) No 576/2006 for animals, the product is depreciated and discarded (Wu, 2004) or used for biogas production (Weiland, 2010; Appels *et al.*, 2011; Nkoa, 2014). The recent European EC Regulation No 2024/1022 further reduced the limit of contamination for food in wheat and wheat products and by‐products for deoxynivalenol (DON), the most common mycotoxin in the small cereal supply chain. Similarly, a downward review of the maximum limits for the feed sector of all *Fusarium* mycotoxins is currently under discussion in Europe.

Faced with this issue, innovative strategies to eliminate mycotoxins from the food chain are required and the use of insects has been proposed as a potential solution (Niermans *et al.*, 2021). During the evolutionary process, the exposure to fungi and their metabolites, leads insects to take advantage of nutrients even when contaminated with mycotoxins. Different degrees of tolerance were observed depending on the insect species and developmental stages. A greater tolerance to aflatoxin B_1_ (AFB_1_) has been observed in *Drosophila melanogaster* Meigen (Diptera: Drosophilidae) eggs and larvae (Llewellyn & Chinnici, 1978). Profound differences in tolerance to AFB_1_ were also observed in larvae of *Amyelois transitella* (Walker) (Lepidoptera: Pyralidae), *Helicoverpa zea* (Boddie) (Lepidoptera: Noctuidae) and *Trichoplusia ni* (Hübner) (Lepidoptera: Noctuidae), showing that the degree of tolerance to AFB_1_ is directly proportional to larval age (Niu *et al.*, 2009; Zeng *et al.*, 2013).

Recently, the possibility to rear insects on mycotoxin‐contaminated substrates has been proposed also for insects suitable for human and livestock consumption (Bisconsin‐Junior *et al.*, 2023). Insects as feed and food can be nutritious, sustainable, and economically viable protein alternatives (Čičková *et al.*, 2015; Van Huis, 2020; Meyer‐Rochow *et al.*, 2022; Van Huis, 2022), especially when associated with the recycling of substrate no longer usable for other purpose. No regulation regarding the feeding of mycotoxin‐contaminated diet to farmed insects is currently available and their use is currently not admitted. Mycotoxin‐contaminated substrates could have a deleterious effect on both the insect development and the final consumer of the insect‐based product. Only few studies are available on the risk of mycotoxin contamination of edible insects (van Broekhoven *et al.*, 2014). Despite several studies have been conducted to determine and quantify the risk of mycotoxin contamination in animal feed there is still a lack of knowledge (van Broekhoven *et al.*, 2017). Research mainly has focused on *Hermetia illucens* (L.) (Diptera: Stratiomyidae) (van Broekhoven *et al.*, 2014; van Broekhoven *et al.*, 2017; Ochoa Sanabria *et al.*, 2019; van Huis, 2022), *Tenebrio molitor* L. (Coleoptera: Tenebrionidae) (van Broekhoven *et al.*, 2014; van Broekhoven *et al.*, 2017; Ochoa Sanabria *et al.*, 2019; Ochoa‐Sanabria, 2019; van Huis, 2022) and *Alphitobius diaperinus* Panzer (Coleoptera: Tenebrionidae) (Ochoa Sanabria *et al.*, 2019) which showed a certain tolerance rate of mycotoxins.

The mycotoxin biotransformation and metabolization has mainly evaluated at the end of the larval stage (Ochoa Sanabria *et al.*, 2019; Ochoa‐Sanabria, 2019) or after a certain period spent on contaminated diet (van Broekhoven *et al.*, 2014; Bosch *et al.*, 2017; Purschke *et al.*, 2018). In most cases the mycotoxin‐excretion rate in exuviae and frass has not been identified cause these fractions are often analyzed together with the diet residues (Camenzuli *et al.*, 2018; Leni *et al.*, 2019). Only in rare cases the effects of mycotoxin have been assessed on multiple life‐stages as in case of *A. diaperinus* larvae, pre‐pupae and beetles exposed to spiked AFB_1_ (Meijer *et al.*, 2022).

As described for AFB_1_ (Bosch *et al.*, 2017) or type B trichothecenes and ochratoxins (Camenzuli *et al.*, 2018), various insect species appear to have different metabolization and excretion pathways for mycotoxins. Therefore, a comprehensive investigation of the metabolism and fate of different mycotoxins in the different insect species is required. Furthermore, since mycotoxins often co‐occur, evaluate accumulation of individual and mixtures of mycotoxins in selected insect species and the maximum level of diet contamination tolerated by insects is crucial (Bosch *et al.*, 2017).

In addition to DON, the most common well‐known mycotoxin which could contaminate cereal grain, there is a great attention to its biologically modified forms, and in particular on the deoxynivalenol‐3‐glucoside (DON‐3‐G), whose chemical modifications, could be introduced by the plant or animal's metabolism (Rychlik *et al.*, 2014). Moreover, mycotoxins such as enniatins (ENNs) and moniliformin (MON), frequently found on cereal matrix, are today reported as emerging mycotoxins, since to date, no regulations exist, and ongoing risk assessment studies are still in progress (Scarpino *et al.*, 2021).

The goals of this study were to determine the effect of the different mycotoxin‐contaminated wheat matrices on *T. molitor* relative growth rate, survival and substrate reduction. Moreover, the capacity of *T. molitor* to accumulate and excrete through exuviae and frass different well‐known and emerging mycotoxins, and their metabolites, was assessed.

## Materials and methods

*Tenebrio molitor* larvae used in the present study originated from mass rearing maintained on a wheat bran diet at the Unit of Entomology of the Department of Agricultural, Forest and Food Sciences (DISAFA; University of Torino, Grugliasco, Italy). Insects were maintained in a climate‐controlled chamber (T: 25 °C; 16 h L : 8 h D photoperiod). Portions of carrots were added twice a week as a source of hydration. Two different trials were set up. The first one aimed to evaluate the insect relative growth rate, survival and substrate reduction while in the second one, the ability of *T. molitor* to accumulate and excrete through exuviae and frass different well‐known and emerging mycotoxins was assessed.

### Tenebrio molitor relative growth rate, survival and substrate reduction

Groups of 300 larvae (IX and X instar) (Park *et al.*, 2014) were collected from the insect mass rearing fed a wheat bran diet. Larvae were weighed and transferred to a plastic tray (24 cm × 17 cm × 12 cm) containing 500 g of diet. Experimental larvae were reared on three different matrices derived from soft wheat (*Triticum aestivum* spp *aestivum*) milling (wholegrain flour, shorts and bran) contaminated with different levels of mycotoxins (Table 1). Experimentally contaminated matrices were obtained by opportunely mixing wheat lots specifically cultivated in the experimental farm of University of Torino located in Carmagnola (Italy) in the 2020 growing season, in order to obtain different mycotoxin contamination at harvest. In particular, wheat grain with an extremely high DON content was obtained by a high‐risk agronomic condition (soil minimum tillage and no fungicide application to protect *Fusarium* infection), while grains with negligible DON content were obtained by a low risk cropping system (ploughing, able of burying previous crop residues, and fungicide application at wheat flowering) (Blandino *et al.*, 2012). From each lot, an aliquot of harvested grains (30 kg) was ground to whole‐meal (wholegrain) using a centrifugal mill equipped with a 1 mm sieve (ETM mill, Vercella Fabio, Mercenasco, Italy), while another aliquot was submitted to multiple‐stream milling by using a roller mill (Bona lab‐scale mill, Labormill 4RB, Monza, Italy) to separate the external coarse bran and the intermediated layers (short) (Pagano *et al.*, 2020). Focusing on the DON, for each of the considered matrices (wholegrain, short, bran), raw materials obtained from the considered agronomic conditions were opportunely and carefully mixed in order to obtain 3 mycotoxin‐contamination levels: low (approximately 700 *µ*g/kg of DON), medium (2800 *µ*g/kg) and high (5600 *µ*g/kg) contamination. This concentration range was defined according to the most extreme contamination values obtainable from all the available naturally contaminated matrices, with the highest and lowest DON content which refers to the bran and the wholegrain obtained by the low‐ and high‐risk agronomic condition, respectively. The level of contamination of each matrix and contamination level for all the considered mycotoxins is reported in Table 1. In total, nine different diets (3 matrices × 3 contamination levels) were assessed. For each diet, three replicates of 300 larvae each were set up. Diet was provided in one solution and slices of carrots were added twice a week as a source of hydration. Insects were maintained in a climate‐controlled chamber (T: 25 °C; 16 h L : 8 h D photoperiod).

**Table 1:** Mycotoxin contamination of wheat matrices used in the experiment

Matrix	Contamination level	DON (*µ*g/kg)	DON‐3‐G (*µ*g/kg)	DON‐3‐G/DON (%)	3‐ADON (*µ*g/kg)	15‐ADON (*µ*g/kg)	DON_TOT_ (*µ*g/kg)	NIV (*µ*g/kg)	ZEA (*µ*g/kg)	ENN_TOT_ (*µ*g/kg)	MON (*µ*g/kg)
Wholegrain	Low	626	67	7.0	70	< LOQ	770	97	78	438	24
	Medium	2844	272	6.2	118	< LOQ	3240	192	387	466	41
	High	5825	575	6.4	190	< LOQ	6596	392	724	772	73
Short	Low	757	78	6.7	41	< LOQ	881	295	93	540	20
	Medium	2755	254	6.0	99	< LOQ	3113	766	213	1340	37
	High	5475	461	5.4	168	< LOQ	6110	1408	392	1901	65
Bran	Low	636	109	11.0	59	< LOQ	809	114	180	873	20
	Medium	2770	311	7.3	126	< LOQ	3212	352	787	1406	30
	High	5767	585	6.6	197	< LOQ	6554	651	1621	2136	43

DON, deoxynivalenol; DON‐3‐G, deoxynivalenol‐3‐glucoside; DON‐3‐G/DON, molar ration of deoxynivalenol‐3‐glucoside and deoxynivalenol; 3‐ADON, 3‐acetyldeoxynivalenol; 15‐ADON, 15‐acetyldeoxynivalenol; DON_TOT_, total deoxynivalenol forms as sum of DON, DON‐3‐G, 3‐ADON and 15‐ADON; NIV, nivalenol; ZEA, zearalenone; ENN_TOT_, total enniatin forms as sum of ENN A, A_1_, B and B_1_; MON, moniliformin; LOQ, limit of quantification = 10 *µ*g/kg.

*Note*: Data are expressed on a dry weight basis.

For each replicate, groups of 100 larvae were weighted every 7 d. For each matrix, the trial was stopped when at least 2 larvae reached the pupal stage and the larval development time was recorded. Larvae were collected, counted and weighed. For each replicate, the average daily gain (ADG) of groups of 100 larvae, the survival rate and the substrate reduction were calculated according to the formulas below:

$$\begin{array}{ccc} \mathrm{ADG} & = & (\mathrm{final}\;\mathrm{weight}-\mathrm{initial}\;\mathrm{weight})/\\ & & \mathrm{number}\;\mathrm{of}\;\mathrm{days}\;\mathrm{on}\;\mathrm{diet}. \end{array}$$

$$\begin{array}{ccc} \mathrm{Survival}\;\mathrm{rate} & = & \left(\mathrm{no}.\mathrm{of}\;\mathrm{alive}\;\mathrm{larvae}/\mathrm{initial}\;\mathrm{no}.\mathrm{of}\;\mathrm{larvae}\right)\\ & & \times 100. \end{array}$$

Substrate reduction (%) = [(initial substrate weight/final substrate weight)/initial substrate weight] × 100 (Scala *et al.*, 2020).

### Mycotoxins levels in Tenebrio molitor

In order to evaluate the capacity of *T. molitor* to accumulate and excrete different mycotoxins, a larger amount of larvae, frass and exuviae were required. With this purpose, larger insect rearings were set up. The same diets used in the previous trial were used. For each tested diet, 100 *T. molitor* adults were collected from the insect mass rearing fed a wheat bran diet and transferred in a plastic box (40 cm × 30 cm). After 7 d of permanence on the tested diet, a necessary time in order to guarantee mating and oviposition, adults were removed. Then, the F1 generation was used in our trials. For each diet, three replicates of 300 larvae each were set up and maintained in a climate‐controlled chamber (T: 25 °C; 16 h L : 8 h D photoperiod).

Substrates were provided *ad libitum* and slices of carrots were added twice a week as a source of hydration. For each matrix, the trial was stopped when at least 2 larvae reached the pupal stage. Alive specimens were grouped (when possible) in batches of 100 larvae each and starved for 24 h in plastic containers (ø 5 cm) closed with a fine mesh net. Then, the frass (from here onwards intended as excrement) was collected. Larvae were washed with sterile water, 75% ethanol, and sterile water for 30 s with the aim of removing any diet residues and any other possible contaminant present in it (e.g., insect frass). Exuviae were periodically collected during the insect rearing. All the collected samples (larvae, exuviae, and frass) were maintained at −20 °C until further analyses.

Ten mycotoxins were searched for at the same time, in each of the analyzed samples: DON, DON‐3‐G, molar ratio of deoxynivalenol‐3‐glucoside and deoxynivalenol (DON‐3‐G/DON), 3‐acetyldeoxynivalenol (3‐ADON), 15‐acetyldeoxynivalenol (15‐ADON), total deoxynivalenol forms (DON_TOT_) as sum of DON, DON‐3‐G, 3‐ADON, and 15‐ADON; nivalenol (NIV); zearalenone (ZEA); total enniatin forms (ENN_TOT_) as sum of ENN A, ENN A_1_, ENN B, and ENN B_1_; moniliformin (MON).

*Tenebrio molitor* larvae (with a 60%−70% of starting humidity) were maintained in a drying oven (MEMMERT GmbH + Co. KG, Schwabach, Germania) at 60 °C for 24 h until a residual humidity of 5%−6% was reached (Purschke *et al.*, 2018; Melgar‐Lalanne *et al.*, 2019). Dried larvae were grounded with a Moulinex chopper (La Moulinette, 800W, Moulinex) and used for humidity‐ and mycotoxin‐level evaluations. Diet samples were directly analyzed due to the lower humidity of the substrates (storage humidity: wholegrain flour 10%, shorts and bran 8%). Due to the small sample size, storage moisture was not assessed for frass and exuviae.

#### Humidity analysis

For each larval and residual matrix sample, a dry weight analysis was performed in order to obtain results that were not dependent on the sample moisture content. Therefore, 3 g of each sample were maintained at 105 °C for 24 h in a laboratory oven (MEMMERT GmbH + Co. KG Schwabach, Germany). After cooling for 15 min, samples were weighed again. The amount of moisture, expressed as a percentage value, was calculated as the difference between the weight of the sample before and after heating at constant temperature with the following formula:

$$\begin{array}{ccc} \mathrm{RH}\left(\%\right) & = & [(\text{fresh weight of the sample}\\ & & -\;\text{dry weight of the sample})\\ & & \times \;\text{fresh weight of the sample}]\times 100. \end{array}$$

#### Multimycotoxin LC‐MS/MS analysis

A 5 g larvae or diet sample was weighted into a 50 mL centrifuge tube and 20 mL of the extracting solution (CH_3_CN/H_2_O/CH_3_COOH 79/20/1, v/v/v) was added. Due to the low quantity of frass and exuviae collected, 0.19−0.20 g of frass and exuviae samples were weighted into a 2 mL or 15 mL centrifuge tube respectively. The extraction solution was added to frass and exuviae samples in the proportion of 1: 4 (w/v) and 1: 20 (w/v) respectively. Extraction and analysis were performed as described in detail by Scarpino *et al.* (2019). Briefly, the extraction was performed for 90 min at 300 r/min using a mechanical shaker (shaker mod. RS‐LS 20, Phoenix Instrument, Garbsen, Germany). The extract obtained from larvae was filtered through Whatman R grade 1 filters (Brentford, United Kingdom) while the extract of frass and exuviae samples was centrifuged at 14 000 r/min for 10 min. The filtered or centrifuged extract were diluted with the same volume of CH_3_CN/H_2_O/CH_3_COOH 20/79/1, v/v/v, vortexed and filtered again through 0.2 *µ*m regenerated cellulose (RC) syringe filters (Phenex‐RC, Phenomenex, Torrance, CA, USA). After appropriate mixing, 20 *µ*L of the diluted filtered extract was injected into the LC‐MS/MS system. Each sample was analyzed twice, both in positive and in negative ionization mode.

LC‐MS/MS analysis was carried out on a Varian 310 triple quadrupole (TQ) mass spectrometer (Varian, Italy), equipped with an electrospray ionization (ESI) source, a 212 LC pump, a ProStar 410 AutoSampler and dedicated software. Liquid chromatography (LC) separation was performed on a Gemini‐NX C18 100 2.1 mm i.d., 5 *µ*m particle size, 110 Å, equipped with a C_18_ 4 mm × 2 mm security guard cartridge column (Phenomenex, Torrance, CA, USA), using water (eluent A) and methanol (eluent B), both acidified with 0.1% v/v CH_3_COOH, as eluents that were delivered at 200 *µ*L/min. The chromatographic and mass spectrometric conditions were described in detail by Scarpino *et al.* (2019). The results pertaining to the linearity range, the limit of detection (LOD), the limit of quantification (LOQ), the apparent recovery R_A_ (%), the matrix effects obtained through the evaluation of the signal suppression/enhancement SSE (%) and the recovery of the extraction R_E_ (%) were reported by Scarpino *et al.* (2019). Following relative humidity correction, the concentration of the different mycotoxins detected was expressed in *µ*g/kg of sample dry matter for larvae or diet sample and in *µ*g/kg of fresh weight for frass and exuviae.

### Statistical analysis

Statistical analyses were performed with SPSS Statistics 28 (SPSS Inc., Chicago, IL, USA) and outcomes were considered significant at *P* < 0.05. The normal distribution and homogeneity of variances were verified by performing the Shapiro–Wilk normality test and the Levene test, respectively. In some analyses, data were transformed, using the *y*’ = ln (*x* + 1) equation, to normalize the residuals. *Tenebrio molitor* relative growth rate, survival, substrate reduction and data on the mycotoxins‐contamination in larvae, exuviae and frass were analyzed by means of a two‐way analysis of variance (ANOVA); in the case of significant differences the means were separated by a Tukey's test. For each combination of matrix × mycotoxins‐contamination level, the mycotoxin content in larvae, exuviae and frass was analyzed by means of a one‐way ANOVA; in the case of significant differences the means were separated by a Tukey's test.

## Results

### Tenebrio molitor relative growth rate, survival, and substrate reduction

The type of wheat matrix significantly influenced the insect relative growth and the diet reduction. The different levels of contamination of the wheat matrices did not lead to significant differences (Table 2). The interaction between wheat matrix and contamination level was significant for the substrate reduction (Table 2).

**Table 2:** Level of significance of the two‐way ANOVA analyses performed to evaluate the contribution of the wheat matrix (*n* = 3, df = 2) and the level of mycotoxin contamination (*n* = 3, df = 2) on larvae parameters and their mycotoxin occurrence (*n* = 9, df = 4). Results are expressed as a percentage of the total mean square

Parameters	Wheat matrix	Contamination level	Matrix × contamination level	Error
Larval development time	74.7^***^	10.6	7.7	7.0
ADG	80.8^***^	8.3	3.8	7.1
Survival rate	33.3	0.00	33.3	33.3
Substrate reduction	66.6^***^	4.6	23.4^*^	5.4
DON	0.3	99.4^***^	0.1	0.1
DON‐3‐G	0.1	99.7^***^	0.1	0.1
DON‐3‐G/DON	4.8	92.8^***^	0.1	2.3
3‐ADON	0.0	99.7^***^	0.2^*^	0.1
DON_TOT_	0.0	99.8^***^	0.1	0.1
NIV	39.5^***^	55.0^***^	5.4^***^	0.1
ZEA	33.0^***^	54.3^***^	12.6^***^	0.1
ENN_TOT_	45.1^***^	51.6^***^	3.2^***^	0.0

DON, deoxynivalenol; DON‐3‐G, deoxynivalenol‐3‐glucoside; DON‐3‐G/DON, molar ratio of deoxynivalenol‐3‐glucoside and deoxynivalenol; 3‐ADON, 3‐acetyldeoxynivalenol; DON_TOT_, total deoxynivalenol forms, sum of DON, DON‐3‐G and 3‐ADON; NIV, nivalenol; ZEA, zearalenone; ENN_TOT_, total enniatin forms, sum of ENN A, A_1_, B and B_1_.

*Note*: Marked factors are statistically significant: *P < 0.05, **P < 0.01, and ***P < 0.001.

The type of matrix used as diet significantly affected the larval development time and the ADG regardless of mycotoxin contamination level (Table 2). A shorter duration of larval development time and a higher ADG was observed in the wholegrain‐based diets (Table 3). Survival rates greater than 91% were observed in all the nine diets tested. Differences due to wheat matrix type and mycotoxin‐contamination level were not observed. The substrate reduction was on average between 5% and 11% in the different contaminated wheat matrices. In rearing fed mycotoxin contaminated wholegrain‐based diet, larger quantities of substrate were consumed in more contaminated diets. Similar substrate reduction values were observed in the shorts and bran diets whatever the level of mycotoxin contamination.

**Table 3:** Effect of wheat matrix and the level of mycotoxin contamination on the larval development time, ADG, survival rate and substrate reduction

Parameter	Wheat matrix	Contamination level			
		Low	Medium	High	Average
Larval development time (d)	Wholegrain	30.67 b	30.33 b	34.33 b	31.77 B
	Shorts	38.33 a	38.67 a	39.67 a	37.89 A
	Bran	41.00 a	34.67 a	38.00 a	38.89 A
	Average	36.67	34.56	37.33	
ADG (g)	Wholegrain	0.0033 a	0.0036 a	0.0031 a	0.0033 A
	Shorts	0.0023 b	0.0022 b	0.0019 b	0.0021 B
	Bran	0.0018 b	0.0026 b	0.0022 b	0.0022 B
	Average	0.0025	0.0028	0.0024	
Survival rate	Wholegrain	91.67% a	95.56% a	95.89% a	94.37%
	Shorts	96.67% a	96.12% a	96.22% a	96.33%
	Bran	94.78% a	95.22% a	93.11% a	94.37%
	Average	94.37%	95.63%	95.07%	
Substrate reduction	Wholegrain	5.27% c	7.34% b	8.52% b	7.05%
	Shorts	8.61% b	9.19% ab	9.50% ab	9.10%
	Bran	10.69% a	9.24% ab	8.61% ab	9.51%
	Average	8.19%	8.59%	8.88%	

*Note*: Within each single factor [wheat matrix (*n* = 3, df = 2) or the contamination level (*n* = 3, df = 2)] means followed by different capital letters are significantly different, according to the Tukey's test; within the values resulting from the combination of matrix level × contamination level (*n* = 9, df = 4), means followed by different lowercase letters are significantly different, according to the Tukey's test. The ANOVA level of significance is shown in Table 2.

### Mycotoxin contamination in larvae, exuviae and frass

A first analysis was carried out to assess whether the degree of mycotoxin accumulation in the larvae could differ according to the wheat matrix contamination level. In all larvae samples, 15‐ADON, a biologically DON modified form, and MON were detected below the limit of quantification. The interaction between wheat matrix and contamination level was significant for 3‐ADON, NIV, ZEA, and ENN_TOT_ (Table 2). The larval accumulation of 7 out of 8 mycotoxins was clearly related to the matrix contamination level. Indeed, a lower accumulation level was always observed in larvae fed a low mycotoxin contaminated matrix and higher accumulation in those fed a high mycotoxin contaminated matrix (Table 4). The same trend of DON was reported also for DON‐3‐G, which is the modified form of the native one, although the DON‐3‐G/DON ratio was higher in the low contamination level compared to high and medium one. If compared to the DON‐3‐G/DON ratio observed in the raw materials (on average 7%) and related to the modification caused by plant metabolism, the ratio detected in the larvae was clearly higher (101%), suggesting a high rate of modification determined by the larval metabolism on the native DON form present in the matrices.

**Table 4:** Effect of wheat matrix and the level of mycotoxin contamination on the contamination of deoxynivalenol (DON) forms and other mycotoxins in insect larvae

Mycotoxin	Wheat matrix	Contamination level			
		Low	Medium	High	Average
DON	Wholegrain	151 c	356 b	441 a	316
	Shorts	145 c	333 b	444 a	308
	Bran	143 c	325 b	431 a	300
	Average	146 C	338 B	439 A	
DON‐3‐G	Wholegrain	266 c	430 b	643 a	449
	Shorts	267 c	431 b	673 a	457
	Bran	274 c	442 b	674 a	463
	Average	269 C	437 B	663 A	
DON‐3‐G/DON	Wholegrain	115 abc	80 d	94 cd	96
	Shorts	119 ab	84 d	98 bcd	100
	Bran	124 a	88 d	101 abcd	105
	Average	120 A	84 C	98 B	
3‐ADON	Wholegrain	5 c	14 b	39 a	20
	Shorts	5 c	15 b	36 a	19
	Bran	5 c	16 b	36 a	19
	Average	5 C	15 B	37 A	
DON_TOT_	Wholegrain	427 c	814 b	1128 a	790
	Shorts	423 c	784 b	1158 a	788
	Bran	428 c	787 b	1146 a	787
	Average	426 C	795 B	1144 A	
NIV	Wholegrain	5 e	18 de	39 c	21 C
	Shorts	27 cd	70 b	135 a	78 A
	Bran	5 e	34 c	70 b	37 B
	Average	13 C	41 B	81 A	
ZEA	Wholegrain	6 ef	14 d	27 b	16 B
	Shorts	5 f	10 de	20 c	12 C
	Bran	8 ef	28 b	74 a	36 A
	Average	7 C	17 B	40 A	
ENN_TOT_	Wholegrain	40 g	47 fg	90 e	59 C
	Shorts	56 f	124 d	165 b	115 B
	Bran	89 e	133 c	175 a	132 A
	Average	61 C	101 B	143 A	

DON, deoxynivalenol; DON‐3‐G, deoxynivalenol‐3‐glucoside; DON‐3‐G/DON, molar ratio of deoxynivalenol‐3‐glucoside and deoxynivalenol; 3‐ADON, 3‐acetyldeoxynivalenol; DON_TOT_, total deoxynivalenol forms, sum of DON, DON‐3‐G and 3‐ADON; NIV, nivalenol; ZEA, zearalenone; ENN_TOT_, total enniatin forms, sum of ENN A, A_1_, B and B_1_.

*Note*: Data are expressed as *µ*g/kg. Within each single factor [wheat matrix (*n* = 3, df = 2) or the contamination level (*n* = 3, df = 2)] means followed by different capital letters are significantly different, according to the Tukey's test; within the values resulting from the combination of matrix level × contamination level (*n* = 9, df = 4), means followed by different lowercase letters are significantly different, according to the Tukey's test. The ANOVA level of significance is shown in Table 2.

**Fig. 1:**
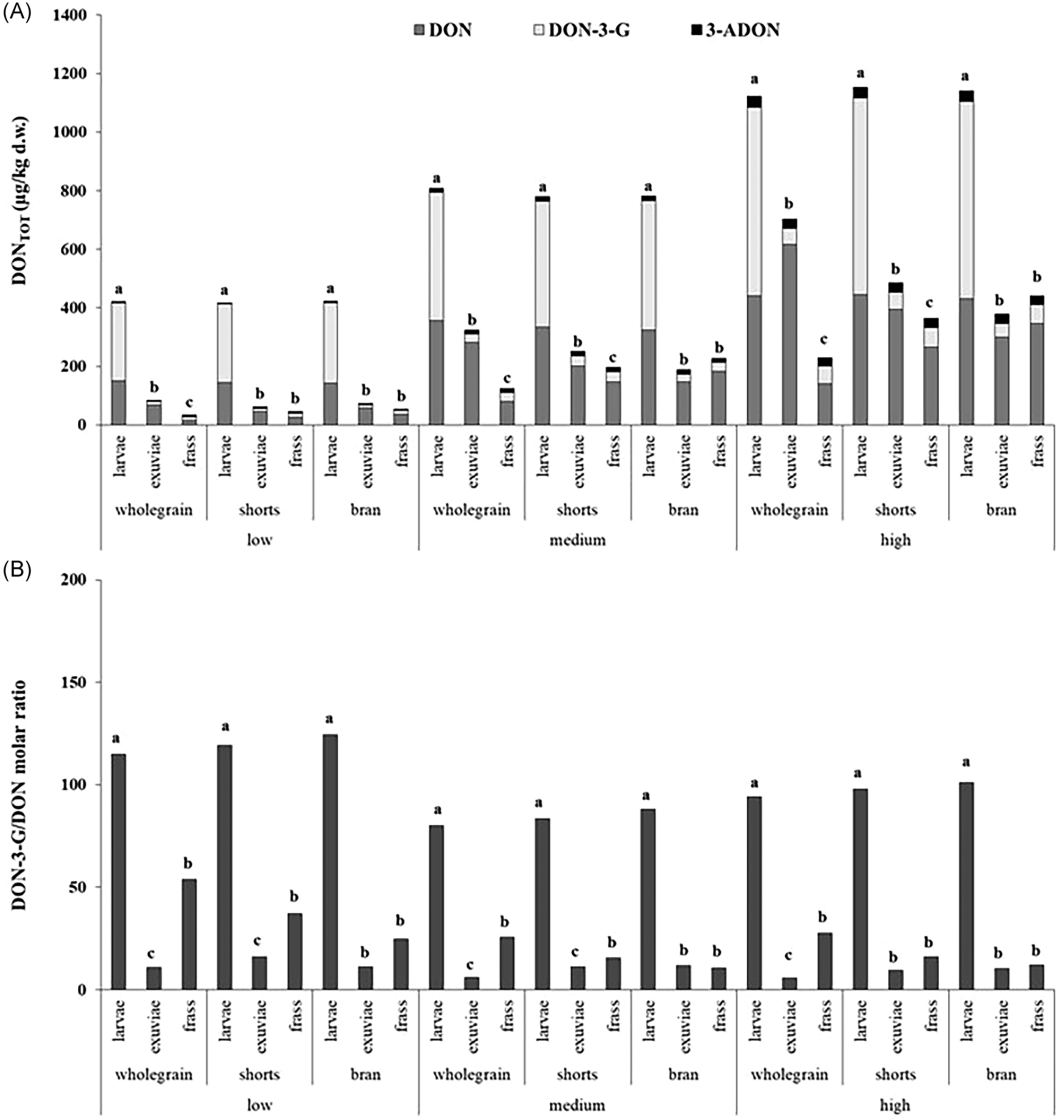
Effects of contamination level for each matrix on the mycotoxin content in larvae, exuviae (periodically collected during the insect rearing) and frass (collected after the 24 h fasting period): (A) total deoxynivalenol content (DON_TOT_, total deoxynivalenol forms, sum of DON, DON‐3‐G and 3‐ADON); (B) molar ration of deoxynivalenol‐3‐glucoside and deoxynivalenol (DON‐3‐G/DON). Within each combination of matrix and mycotoxin level, different letters above the bars indicate significant differences between larvae, exuviae and frass, according to the Tukey's test. The ANOVA level of significance is shown in Table 2.

The type of matrix did not affect DON and modified forms, or DON‐3‐G/DON ratio, while they significantly influenced the levels of NIV, ZEA, and ENN_TOT_ accumulation in the larvae (Table 4). In accordance with the different contamination levels observed in the matrices for these mycotoxins, which are distributed in the by‐products of milling processes differently than DON, the highest NIV accumulation values were detected in shorts, while the highest ZEA and ENN_TOT_ accumulation level were recorded in bran. Similarly to the results observed for larvae, the contamination of all the mycotoxins also increased proportionally in exuviae (Table S2) and frass (Table S3) in accordance with the content in the matrix used in the diet. More variable effects were reported as far as the type of matrix is concerned: the exuviae derived from the wholegrain diet reported the highest DON content, while for frass the concentration was significantly higher in the bran diet. According to the higher concentration in the raw materials, ZEA and ENN_TOT_ accumulation were more concentrated in exuviae and frass collected from insect fed with bran. Afterwards, analyses were conducted in order to assess the different level of mycotoxins accumulation in larvae and their ability to expel them through exuviae and frass. Significant differences in the DON_TOT_ accumulation level among larvae, exuviae and frass were observed for all the tested matrices (Fig. 1A). Higher accumulation levels were detected when insects fed a higher mycotoxins‐contaminated matrices. For all the wheat matrices and contamination levels, a higher accumulation of DON was always observed in larvae except for the wholegrain contaminated with high DON level where a highest accumulation level was observed in exuviae (Fig. 1A). As far as the modified DON forms are concerned, DON‐3‐G and 3‐ADON were on average 32% and 6% of the total DON, respectively. The 15‐ADON was never detected also in the exuviae and frass. Regardless of the matrix used, a higher level of DON‐3‐G accumulation was always observed in larvae and a lower in exuviae (Fig. 1A). Similar levels of 3‐ADON accumulation in larvae, exuviae and frass were observed when insects fed a matrix contaminated with low DON levels. The highest levels of 3‐ADON accumulation were observed in larvae fed on matrices contaminated with high DON levels and the bran contaminated with medium DON level. In the remaining cases (wholegrain and shorts with medium level of DON contamination), a higher 3‐ADON accumulation was observed in exuviae (Fig. 1A). The percentage of DON and total DON forms in the larvae, exuviae and frass compared to the level in the substrate matrix is reported in Table S4. For each matrix the DON values were always higher in larvae (14%) compared to exuviae (8%) and frass (5%), while the percentage decreases as the level of contamination of the substrate increases.

A higher DON‐3‐G/DON ratio was always observed in larvae, generally followed by frass and exuviae (Fig. 1B). In accordance with the highest native DON occurrence, the differences between larvae, exuviae and frass in the DON‐3‐G/DON ratio were reduced when moving from low to high mycotoxin contamination levels in the matrices.

**Fig. 2:**
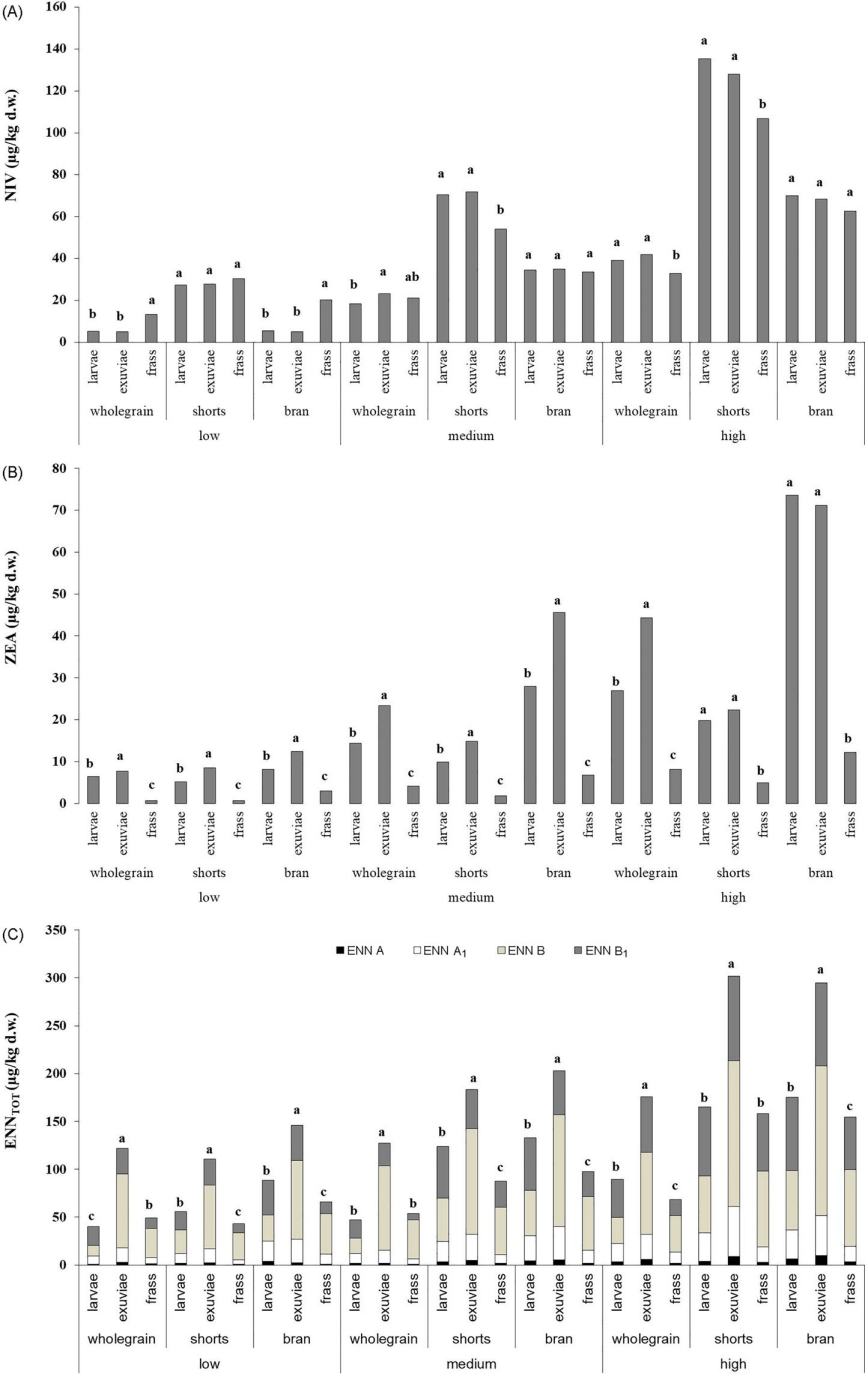
Effects of contamination level for each matrix on the mycotoxin content in larvae, exuviae (periodically collected during the insect rearing) and frass (collected after the 24 h fasting period): (A) nivalenol (NIV); (B) zearalenone (ZEA); (C) total enniatin content (ENN_TOT_, sum of ENN A, ENN A_1_, ENN B, and ENN B_1_). Within each combination of matrix and mycotoxin level, different letters above the bars indicate significant differences between larvae, exuviae, and frass, according to the Tukey's test. The ANOVA level of significance is shown in Table 2.

For bran, differences in NIV accumulation were only observed when insects fed a diet contaminated with low mycotoxin contamination when higher accumulation values were detected in frass. Contaminated shorts with higher levels of mycotoxin led to significant accumulation in larvae and exuviae. The wholegrains matrix showed a more variable behaviour, as a function of the different contamination levels. This behaviour is linked to a greater accumulation in exuviae and larvae of insects fed a higher contaminated diet (Fig. 2A). Higher values of ZEA accumulation were detected in exuviae followed by larvae and frass with the exception of shorts and bran contaminated with high levels of mycotoxin. In these cases, similar values have been observed in larvae and exuviae. On average, ENN A, ENN A_1_, ENN B, and ENN B_1_ accounted respectively for 3%, 16%, 51%, and 31% of the ENN_TOT_ contamination level. ENN_TOT_ were generally accumulated in exuviae followed by larvae and frass. Only in insects fed a wholegrain diet contaminated with low mycotoxin level, the accumulation recorded in frass was higher compared to the one observed in larvae.

## Discussion

The larva of *T. molitor* represents a new sustainable source of protein and nutrients for animal and human nutrition. Its rearing shows great promise in the rapidly expanding, global, edible insect market, where it is already being widely produced as feed for fish and poultry (Thévenot *et al.*, 2018; Sogari *et al.*, 2019; Selaledi *et al.*, 2020). Although there are still concerns regarding the possible use of mycotoxins‐contaminated substrates as a rearing diet for edible insects, it is interesting to evaluate their possible feasibility. To ensure safe insect‐based feeds or foods, the risks of mycotoxins and other secondary metabolites must be elucidated. The behavioral and physiological effect of mycotoxins in insect has been poorly investigated so far, especially in the larval stage of insects (Janković‐Tomanić *et al.*, 2023), and toxicological research to fully understand the safe limits of mycotoxins for insect health in large‐scale rearing is required (Camenzuli *et al.*, 2018). Several aspects need to be clarified concerning mycotoxins and their metabolites impact on the insect immune system. To our knowledge, just one study has examined this last aspect. The administration of an AFB_1_‐spiked diet in *H. illucens* rearing resulted in an upregulation of genes involved in AFB_1_ metabolism and in the downregulation of genes generally involved in the insect growth, development, and immunity. This is indicative of a trade‐off between detoxification and immune responses (Shah *et al.*, 2024) and should be further deeply investigated.

In our study, insects successfully metabolize mycotoxins without any negative consequences on their survival as shown also by other researchers (Van Broekhoven *et al.*, 2017; Ochoa Sanabria *et al.*, 2019). Our results demonstrated that after fed matrices contaminated with different wheat mycotoxin levels there was no negative impact on insect relative growth rate. As expected, the type of wheat matrix influenced the larval development time and the ADG. A shorter development time (up to 11 d) and a higher ADG was obtained in insect fed wholegrain‐based diets due to their higher nutritional value (Cohen, 2015). Lower substrate consumption was observed in the wholegrain matrix presumably due to its nutritive profile, which allows larvae to have a more efficient feeding. Only for this matrix, the substrate reduction was influenced by the level of mycotoxins‐contamination. In particular, a higher consumption was recorded in a diet contaminated with medium and high levels of mycotoxins. A first explanation could be related to the lower relative starch content, and therefore of the energy value, of kernels subjected to a high *Fusarium* Head Blight attack in field, which reduced the test weight, and thus the endosperm/pericarp ratio, compared to wheat cultivated with a low disease pressure (Siuda *et al.*, 2010). Moreover, it has already been proved that *T. molitor* is more attracted by fungi‐contaminated substrates than uncontaminated diets (Guo *et al.*, 2014; Van Broekhoven *et al.*, 2017). However, due to increased mycotoxin‐toxicity, higher contamination level may affect the insect food intake in order to compensate for its physiological/immunological efforts. For example, it is possible that under these circumstances more energy is required for the defense mechanisms.

Our research confirms that mycotoxins and their secondary metabolites do not bioaccumulate in the insect bodies (Leni *et al.*, 2019) and well highlights the ability of *T. molitor* to excrete mycotoxins through the exuviae. To the best of our knowledge, this is the first study that quantifies this capacity in detail and for several mycotoxins. The lack of information in literature may be due to the difficulty in collecting a sufficient amount of exuviae for the analysis. This insect species, compared to others, performs numerous larval molts (10−20) and is therefore able to excrete a high quantity of ingested mycotoxins. In our trials collecting all exuviae and frass in purity during the entire larval development was impracticable. Therefore, our results concerning the ability to excrete mycotoxins, through the exuviae and the frass, could most likely be underestimated.

According with Ochoa Sanabria *et al.* (2019), DON‐ and DON‐3‐G content in larvae showed to be related to the matrix‐contamination level. Indeed, higher quantities of both DON and DON‐3‐G, its glucosidated forms, have been observed in insect fed a diet contaminated with higher mycotoxin level. Contrary to what reported by Van Broekhoven *et al.* (2017), we detected DON derivatives in larvae. A higher DON‐3‐G level was detected in larvae than in the starting substrates, demonstrating the active role of larvae in metabolizing DON into DON‐3‐G and accumulating it in this form. Interestingly, we detected higher DON‐3‐G/DON ratio in larvae feeding on substrates with a low levels of contamination rather than those with a high contamination levels. The inverse relationship between the level of contamination of DON in the matrix and the DON‐3‐G/DON ratio in the larvae was similar to previous studies focusing in in planta quantification of DON‐3‐G (Scarpino & Blandino, 2021), who suggested that the plant process of DON conjugation to glucose occurs with an intensity that is barely influenced by the occurrence of the free mycotoxin form, but which instead appears closely connected to the metabolic rate and the threshold biosynthetic ability of the plant. Similarly, in our trials carried out on matrices with high levels of mycotoxins contamination, the metabolic activity of the larvae does not lag behind the DON availability, and therefore the modified fraction is reduced from the total.

Although many studies have investigated insect tolerance to mycotoxins and the produced metabolites, few studies regarding the metabolization of mycotoxins in insects are available (Evans & Shao, 2022). The role of a phase II enzyme glucosyltransferase was proposed to be involved in the detoxification of DON into DON‐3‐G in aphids (De Zutter *et al.*, 2016). To date, no information is available on detoxification of DON or other mycotoxins by Coleoptera (Niermans *et al.*, 2021). Further research needs to be conducted to understand the exact enzymes and pathways that are involved. Although some of the DON‐3‐G considered in our analysis could have been due to the metabolism of the plant used as rearing substrate, our results underline that *T. molitor* poorly accumulates the mycotoxin it ingests (DON), but essentially accumulates the toxins it successfully modifies (DON‐3‐G).

Similar levels of 3‐ADON were observed among exuviae, frass, and larvae fed diets contaminated with low and medium levels of mycotoxin. A greater accumulation was always recorded in larvae reared on the high DON level (wholegrain: 39 *µ*g/kg; shorts and bran: 36 *µ*g/kg). Through the exuviae and frass the insects excreted about twice the mycotoxin content than in the larvae independently from the matrix and the diet contamination level.

In our trials, the 35%, 43%, and 39% of DON_TOT_ was detected in the form of DON in larvae fed a low, medium and high level of mycotoxins‐contaminated diet respectively. The 64%, 55% and 58% of the DON_TOT_ was recorded as DON‐3‐G in the larvae, while the 3‐ADON was detected up to 3%. It is interesting to point out that these proportions drastically change in exuviae and frass. The 78%, 82%, 83%, and 56%, 73% and 71% of DON_TOT_ was detected in the form of DON in exuviae and frass excreted by insect fed a low, medium and high level of mycotoxins‐contaminated diet respectively. This indicates that most of the mycotoxins is excreted in unaltered state, although a quote, which proportionally decreased according to the availability of native DON form in the matrix, is metabolized by the insect and accumulated in the larvae body. In insects fed a wholegrain diet contaminated with high mycotoxin levels, DON was mainly excreted through exuviae (617 *µ*g/kg).

Contrary to what was reported by other authors (Guo *et al.*, 2014; Van Broekhoven *et al.*, 2017), DON and its derivatives (DON‐3‐G and 3‐ADON) were detected in all the analyzed larvae. As already pointed out by Ochoa Sanabria *et al.* (2019), this could be due to the longer time spent on the contaminated substrates compared to the 15 d reported by the other authors. Moreover, modulation in the diet could also lead to changes in the gut microbiota in larvae and facilitate the transformation of DON (Ochoa Sanabria *et al.*, 2019). Indeed, the interaction between intestinal enzyme activity and intestinal microflora facilitates these processes (Genta *et al.*, 2006). This also explain why DON‐3‐G level was found in larvae in higher concentrations than those in the starting substrates. Compared with DON, DON‐3‐G shows lower toxicity to humans and is not absorbed as such after oral ingestion by monogastric species. However, in polygastric animals it is biotransformed into DON when it is ingested into the digestive tract (Poppenberger *et al.*, 2003; Galaverna *et al.*, 2009; Nagl *et al.*, 2014; Broekaert *et al.*, 2016, 2017; Pierron *et al.*, 2016; Vidal *et al.*, 2018). Notably, DON‐3‐G is generally not hydrolyzed to DON in chickens (Sun *et al.*, 2022) thus allowing *T. molitor* larvae with a higher content of this metabolite to be destined for their feeding.

ZEA was detected in the larvae grown in all the matrices and mycotoxin contamination levels. The highest value (73.5 *µ*g/kg) was recorded in larvae fed with high‐mycotoxin‐contaminated bran. As already observed for *A. diaperinus* and for *H. illucens* (Camenzuli *et al.*, 2018), *T. molitor* excrete most of this ingested mycotoxin through exuviae. Only in larvae fed a shorts‐ and bran‐based diet the amount of ZEA detected in the larvae was very similar to that excreted with the exuviae. From the toxicological point of view, particular attention should be paid to ZEA derivatives. We do not investigate this aspect, however the presence of *α*‐ZEAol and *β*‐ZEAol has been previously detected in excrements (Niermans *et al.*, 2019) confirming that detoxification enzymes are present in *T. molitor*, which makes ZEA metabolism in larvae possible (Kostaropoulos *et al.*, 1996).

In our trials, the NIV content in larvae, exuviae and frass showed to be related to the matrix‐contamination level. Similar levels of NIV have been observed in larvae, exuviae and frass, demonstrating *T. molitor* ability to excrete it. Little is known about the metabolic processes involved in the degradation of NIV. Anyhow, since this mycotoxin is a trichothecene, its degradation may be mediated by mechanisms similar to those involved during DON detoxification.

This is the first research concerning the occurrence of MON and ENN_TOT_ in insects. In all analyses, MON was below the limit of quantification (1 *µ*g/kg). This result may be due to its molecular characteristics (molecule with a simple structure and therefore rapid degradation, Ferrigo *et al.*, 2021), although more researches are necessary in order to verify this assumption, also considering raw material with higher concentration of this mycotoxin compared to the level considered in the present experiment. On the contrary, our trials prove the ability of *T. molitor* to excrete ENN_TOT_ through the exuviae. Indeed, we measured up to three times more ENN_TOT_ in the exuviae than in the larvae. On average, ENN A, ENN A_1_, ENN B, ENN B_1_ accounted respectively for 3%, 16%, 51%, and 31% of the ENN_TOT_ contamination level. Interestingly, the ratio between the forms of ENNs was changed by the metabolic activity of the insect. Indeed, on average, in larvae ENN A, ENN A_1_, ENN B, ENN B_1_ accounted respectively for 3%, 20%, 35%, and 42% of the ENN_TOT_ contamination level, while, in the starting matrices with which the larvae were fed, they on average accounted respectively for 2%, 10%, 69%, and 19%.

Ultimately, our results demonstrated that after consuming high concentrations of the different mycotoxins for their entire growth, the excretion through exuviae and frass resulted in larvae with overall lower levels of mycotoxins. In accordance with the contamination levels of the raw materials compared in the present study, which determine potential constraints on their use in feed, in particular for some animal categories (Table S1), the levels obtained in the larvae are always lower than those permitted for human and animal consumption. In fact, the level of contamination of DON_TOT_ and ZEA in the larvae fed with the high level of contamination of the wholegrain matrix was respectively reduced by 83% and 96%, compared to the starting matrix. These results open up new scenarios on the possibility of large‐scale rearing of *T. molitor* for livestock consumption on mycotoxin‐contaminated substrates. Currently, the use of mycotoxins‐contaminated substrates for insect farming is not yet regulated in Europe, but the safety limits for these metabolites could be similar to or higher than the current limits for production animals.

## Disclosure

The authors declare that they have no conflict of interest.

## Supporting information




**Table S1** Guideline values for deoxynivalenol, zearalenone, ochratoxin A, fumonisins B_1_+B_2_, and T‐2 and HT‐2 toxin in feed materials and compound feed set by Commission Recommendation 2006/576/EC of August 17, 2006.
**Table S2** Effect of wheat matrix and the level of mycotoxin contamination on the contamination of deoxynivalenol form and other mycotoxins in insect exuviae.
**Table S3** Effect of wheat matrix and the level of mycotoxin contamination on the contamination of deoxynivalenol form and other mycotoxins in insect frass.
**Table S4** Content of deoxynivalenol and total deoxynivalenol forms in insect larvae, exuviae, and frass expressed as percentage of the concentration occurred in the wheat matrix used for feeding the insects.

## References

[ins70066-bib-0001] Appels, L. , Lauwers, J. , Degrève, J. , Helsen, L. , Lievens, B. , Willems, K. *et al.* (2011) Anaerobic digestion in global bio‐energy production: potential and research challenges. Renewable and Sustainable Energy Reviews, 15, 4295–4301.

[ins70066-bib-0002] Bisconsin‐Junior, A. , Feitosa, B.F. , Silva, F.L. and Mariutti, L.R.B. (2023) Mycotoxins on edible insects: should we be worried? Food and Chemical Toxicology, 177, 113845. 37209938 10.1016/j.fct.2023.113845

[ins70066-bib-0003] Blandino, M. , Haidukowski, M. , Pascale, M. , Plizzari, L. , Scudellari, D. and Reyneri, A. (2012) Integrated strategies for the control of *Fusarium* head blight and deoxynivalenol contamination in winter wheat. Field Crops Research, 133, 139–149.

[ins70066-bib-0004] Bosch, G. , van der Fels‐Klerx, H.J. , de Rijk, T.C. and Oonincx, D.G.A.B. (2017) Aflatoxin B1 tolerance and accumulation in black soldier fly larvae (*Hermetia illucens*) and yellow mealworms (*Tenebrio molitor*). Toxins, 9, 185. 28574433 10.3390/toxins9060185 PMC5488035

[ins70066-bib-0005] Broekaert, N. , Devreese, M. , Demeyere, K. , Berthiller, F. , Michlmayr, H. , Varga, E. *et al.* (2016) Comparative in vitro cytotoxicity of modified deoxynivalenol on porcine intestinal epithelial cells. Food and Chemical Toxicology, 95, 103–109. 27338712 10.1016/j.fct.2016.06.012

[ins70066-bib-0006] Broekaert, N. , Devreese, M. , van Bergen, T. , Schauvliege, S. , De Boevre, M. , De Saeger, S. *et al.* (2017) *In vivo* contribution of deoxynivalenol‐3‐*β*‐D‐glucoside to deoxynivalenol exposure in broiler chickens and pigs: oral bioavailability, hydrolysis and toxicokinetics. Archives of Toxicology, 91, 699–712. 27100115 10.1007/s00204-016-1710-2

[ins70066-bib-0007] Camenzuli, L. , Van Dam, R. , de Rijk, T. , Andriessen, R. , Van Schelt, J. , Van Der Fels‐Klerx, H. *et al.* (2018) Tolerance and excretion of the mycotoxins aflatoxin B1, zearalenone, deoxynivalenol, and ochratoxin A by *Alphitobius diaperinus* and *Hermetia illucens* from contaminated substrates. Toxins, 10, 91. 29495278 10.3390/toxins10020091 PMC5848191

[ins70066-bib-0008] Cohen, A.C. (2015) Insect Diets: Science and Technology (2nd edn). Routledge & CRC Press, Boca Raton.

[ins70066-bib-0009] Čičková, H. , Newton, G.L. , Lacy, R.C. and Kozánek, M. (2015) The use of fly larvae for organic waste treatment. Waste Management, 35, 68–80. 25453313 10.1016/j.wasman.2014.09.026

[ins70066-bib-0010] De Zutter, N. , Audenaert, K. , Arroyo‐Manzanares, N. , De Boevre, M. , Van Poucke, C. , De Saeger, S. *et al.* (2016) Aphids transform and detoxify the mycotoxin deoxynivalenol via a type II biotransformation mechanism yet unknown in animals. Scientific Reports, 6, 38640. 27929076 10.1038/srep38640 PMC5144147

[ins70066-bib-0011] Evans, N.M. and Shao, S. (2022) Mycotoxin metabolism by edible insects. Toxins, 14, 217. 35324714 10.3390/toxins14030217 PMC8949902

[ins70066-bib-0012] FAO (2003) Worldwide regulations for mycotoxins in food and feed in 2003. https://www.fao.org/4/y5499e/y5499e00.htm

[ins70066-bib-0013] Ferrigo, D. , Scarpino, V. , Vanara, F. , Causin, R. , Raiola, A. and Blandino, M. (2021) Influence of H₂O₂‐induced oxidative stress on in vitro growth and moniliformin and fumonisins accumulation by *Fusarium proliferatum* and *Fusarium subglutinans* . Toxins, 13, 653. 34564657 10.3390/toxins13090653 PMC8473447

[ins70066-bib-0014] Galaverna, G. , Dall'Asta, C. , Mangia, M. , Dossena, A. and Marchelli, R. (2009) Masked mycotoxins: an emerging issue for food safety. Czech Journal of Food Sciences, 27, S89–S92.

[ins70066-bib-0015] Genta, F.A. , Dillon, R.J. , Terra, W.R. and Ferreira, C. (2006) Potential role for gut microbiota in cell wall digestion and glucoside detoxification in *Tenebrio molitor* larvae. Journal of Insect Physiology, 52, 593–601. 16600286 10.1016/j.jinsphys.2006.02.007

[ins70066-bib-0016] Guo, Z. , Döll, K. , Dastjerdi, R. , Karlovsky, P. , Dehne, H.‐W. and Altincicek, B. (2014) Effect of fungal colonization of wheat grains with *Fusarium* spp. on food choice, weight gain and mortality of meal beetle larvae (*Tenebrio molitor*). PLoS ONE, 9, e100112. 24932485 10.1371/journal.pone.0100112 PMC4059719

[ins70066-bib-0017] Janković‐Tomanić, M. , Petković, B. , Vranković, J. and Perić‐Mataruga, V. (2023) Changes in antioxidant enzymes and locomotor activity of yellow mealworm larvae fed the mycotoxin Zearalenone supplemented diet. Journal of Stored Products Research, 102, 102113.

[ins70066-bib-0018] Kostaropoulos, I. , Mantzari, A.E. and Papadopoulos, A.I. (1996) Alterations of some glutathione S‐transferase characteristics during the development of *Tenebrio molitor* (Insecta: Coleoptera). Insect Biochemistry and Molecular Biology, 26, 963–969.

[ins70066-bib-0019] Leni, G. , Cirlini, M. , Jacobs, J. , Depraetere, S. , Gianotten, N. , Sforza, S. *et al.* (2019) Impact of naturally contaminated substrates on *Alphitobius diaperinus* and *Hermetia illucens*: uptake and excretion of mycotoxins. Toxins, 11, 476. 31426582 10.3390/toxins11080476 PMC6722799

[ins70066-bib-0020] Llewellyn, G.C. and Chinnici, J.P. (1978) Variation in sensitivity to aflatoxin B1 among several strains of *Drosophila melanogaster* (Diptera). Journal of Invertebrate Pathology, 31, 37–40. 415091 10.1016/0022-2011(78)90106-4

[ins70066-bib-0021] Medina, Á. , González‐Jartín, J.M. and Sainz, M.J. (2017) Impact of global warming on mycotoxins. Current Opinion in Food Science, 18, 76–81.

[ins70066-bib-0022] Meijer, N. , Nijssen, R. , Bosch, M. , Boers, E. and van der Fels‐Klerx, H.J. (2022) Aflatoxin B1 metabolism of reared *Alphitobius diaperinus* in different life‐stages. Insects, 13, 357. 35447799 10.3390/insects13040357 PMC9025786

[ins70066-bib-0023] Melgar‐Lalanne, G. , Hernández‐Álvarez, A.J. and Salinas‐Castro, A. (2019) Edible insects processing: traditional and innovative technologies. Comprehensive Reviews in Food Science and Food Safety, 18, 1166–1191. 33336989 10.1111/1541-4337.12463

[ins70066-bib-0024] Meyer‐Rochow, V.B. , Pinent, M. , Costa Neto, E.M. , Grabowski, N.T. , Fratini, F. and Mancini, S. (2022) Editorial: insects as food and feed. Frontiers in Veterinary Science, 9, 873765. 35425834 10.3389/fvets.2022.873765 PMC9001884

[ins70066-bib-0025] Moretti, A. , Pascale, M. and Logrieco, A.F. (2019) Mycotoxin risks under a climate change scenario in Europe. Trends in Food Science & Technology, 84, 38–40.

[ins70066-bib-0026] Nagl, V. , Woechtl, B. , Schwartz‐Zimmermann, H.E. , Hennig‐Pauka, I. , Moll, W.D. , Adam, G. *et al.* (2014) Metabolism of the masked mycotoxin deoxynivalenol‐3‐glucoside in pigs. Toxicology Letters, 229, 190–197. 24968060 10.1016/j.toxlet.2014.06.032

[ins70066-bib-0027] Niermans, K. , Woyzichovski, J. , Kröncke, N. , Benning, R. and Maul, R. (2019) Feeding study for the mycotoxin zearalenone in yellow mealworm (*Tenebrio molitor*) larvae—investigation of biological impact and metabolic conversion. Mycotoxin Research, 35, 231–242. 30864055 10.1007/s12550-019-00346-y PMC6611894

[ins70066-bib-0028] Niermans, K. , Meyer, A.M. , den Hil, E.F.H. , Van Loon, J.J.A. and van der Fels‐Klerx, H.J. (2021) A systematic literature review on the effects of mycotoxin exposure on insects and on mycotoxin accumulation and biotransformation. Mycotoxin Research, 37, 279–295. 34618340 10.1007/s12550-021-00441-z PMC8571154

[ins70066-bib-0029] Niu, G. , Siegel, J. , Schuler, M.A. and Berenbaum, M.R. (2009) Comparative toxicity of mycotoxins to navel orangeworm (*Amyelois transitella*) and corn earworm (*Helicoverpa zea*). Journal of Chemical Ecology, 35, 951–957. 19680726 10.1007/s10886-009-9675-8

[ins70066-bib-0030] Nkoa, R. (2014) Agricultural benefits and environmental risks of soil fertilization with anaerobic digestates: a review. Agronomy for Sustainable Development, 34, 473–492.

[ins70066-bib-0031] Ochoa‐Sanabria, C. (2019) Feeding mycotoxin‐contaminated wheat to insect larvae (*Tenebrio molitor*) as a strategy to use *Fusarium*‐damaged grain and generate a safe protein replacement for animal feed. Bulletin de la Société D'entomologie du Canada, 50, 31–35.

[ins70066-bib-0032] Ochoa Sanabria, C. , Hogan, N. , Madder, K. , Gillott, C. , Blakley, B. and Reaney, M.J.T. (2019) Yellow mealworm larvae (*Tenebrio molitor*) fed mycotoxin‐contaminated wheat—a possible safe, sustainable protein source for animal feed? Toxins, 11, 282. 31117211 10.3390/toxins11050282 PMC6563207

[ins70066-bib-0033] Pagani, M.A. , Giordano, D. , Cardone, G. , Pasqualone, A. , Casiraghi, M.C. , Erba, D. *et al.* (2020) Nutritional features and bread‐making performance of whole wheat: Does the milling system matter? Foods, 9, 1035. 32752209 10.3390/foods9081035 PMC7466235

[ins70066-bib-0034] Park, J.B. , Choi, W.H. , Kim, S.H. , Jin, H.J. , Han, Y.S. , Lee, Y.S. *et al.* (2014) Developmental characteristics of *Tenebrio molitor* larvae (Coleoptera: Tenebrionidae) in different instars. International Journal of Industrial Entomology, 28, 5–9.

[ins70066-bib-0035] Pierron, A. , Mimoun, S. , Murate, L.S. , Loiseau, N. , Lippi, Y. , Bracarense, A.‐P.F.L. *et al.* (2016) Intestinal toxicity of the masked mycotoxin deoxynivalenol‐3‐*β*‐d‐glucoside. Archives of Toxicology, 90, 2037–2046. 26404761 10.1007/s00204-015-1592-8

[ins70066-bib-0036] Poppenberger, B. , Berthiller, F. , Lucyshyn, D. , Sieberer, T. , Schuhmacher, R. , Krska, R. *et al.* (2003) Detoxification of the *Fusarium* mycotoxin deoxynivalenol by a UDP‐glucosyltransferase from *Arabidopsis thaliana* . The Journal of Biological Chemistry, 278, 47905–47914. 12970342 10.1074/jbc.M307552200

[ins70066-bib-0037] Purschke, B. , Brüggen, H. , Scheibelberger, R. and Jäger, H. (2018) Effect of pre‐treatment and drying method on physico‐chemical properties and dry fractionation behaviour of mealworm larvae (*Tenebrio molitor* L.). European Food Research and Technology, 244, 269–280.

[ins70066-bib-0038] Rao, V.K. , Shilpa, P. , Girisham, S. and Reddy, S.M. (2011) Incidence of mycotoxigenic penicillia in feeds of Andhra Pradesh, India. Journal of Mycotoxins and Plant Pathogens, 2, 46–50.

[ins70066-bib-0039] Rychlik, M. , Humpf, H.U. , Marko, D. , Dänicke, S. , Mally, A. , Berthiller, F. *et al.* (2014) Proposal of a comprehensive definition of modified and other forms of mycotoxins including “masked” mycotoxins. Mycotoxin Research, 30, 197–205. 24962446 10.1007/s12550-014-0203-5 PMC4202116

[ins70066-bib-0040] Scala, A. , Cammack, J.A. , Salvia, R. , Scieuzo, C. , Franco, A. , Bufo, S.A. *et al.* (2020) Rearing substrate impacts growth and macronutrient composition of *Hermetia illucens* (L.) (Diptera: Stratiomyidae) larvae produced at an industrial scale. Scientific Reports, 10, 19448. 33173088 10.1038/s41598-020-76571-8 PMC7655861

[ins70066-bib-0057] Scarpino, V. and Blandino, M. (2021) Effects of durum wheat cultivars with different degrees of FHB susceptibility grown under different meteorological conditions on the contamination of regulated, modified and emerging mycotoxins. Microorganisms, 9(2), 408. 10.3390/microorganisms9020408 33669359 PMC7920256

[ins70066-bib-0041] Scarpino, V. , Reyneri, A. and Blandino, M. (2019) Development and comparison of two multiresidue methods for the determination of 17 *Aspergillus* and *Fusarium* mycotoxins in cereals using HPLC‐ESI‐TQ‐MS/MS. Frontiers in Microbiology, 10, 361. 30886605 10.3389/fmicb.2019.00361 PMC6409351

[ins70066-bib-0042] Scarpino, V. , Vanara, F. , Sulyok, M. , Krska, R. and Blandino, M. (2021) Fate of regulated, masked, emerging mycotoxins and secondary fungal metabolites during different large‐scale maize dry‐milling processes. Food Research International, 140, 109861. 33648179 10.1016/j.foodres.2020.109861

[ins70066-bib-0043] Selaledi, L. , Mbajiorgu, C.A. and Mabelebele, M. (2020) The use of yellow mealworm (*T. molitor*) as alternative source of protein in poultry diets: a review. Tropical Animal Health and Production, 52, 7–16. 31392553 10.1007/s11250-019-02033-7

[ins70066-bib-0044] Shah, P.N. , Niermans, K. , Hoek‐van den Hil, E.F. , Dicke, M. and van Loon, J.J.A. (2024) Effects of Aflatoxin B1 on metabolism‐ and immunity‐related gene expression in *Hermetia illucens* L. (Diptera: Stratiomyidae). Pesticide Biochemistry and Physiology, 202, 105944. 38879301 10.1016/j.pestbp.2024.105944

[ins70066-bib-0045] Siuda, R. , Grabowski, A. , Lenc, L. , Ralcewicz, M. and Spychaj‐Fabisiak, E. (2010) Influence of the degree of fusariosis on technological traits of wheat grain. International Journal of Food Science & Technology, 45, 2596–2604.

[ins70066-bib-0046] Sogari, G. , Amato, M. , Biasato, I. , Chiesa, S. and Gasco, L. (2019) The potential role of insects as feed: a multi‐perspective review. Animals, 9, 119. 30934748 10.3390/ani9040119 PMC6523843

[ins70066-bib-0047] Sun, Y. , Jiang, J. , Mu, P. , Lin, R. , Wen, J. and Deng, Y. (2022) Toxicokinetics and metabolism of deoxynivalenol in animals and humans. Archives of Toxicology, 96, 2639–2654. 35900469 10.1007/s00204-022-03337-8

[ins70066-bib-0048] Thévenot, A. , Rivera, J.L. , Wilfart, A. , Maillard, F. , Hassouna, M. , Senga‐Kiesse, T. *et al.* (2018) Mealworm meal for animal feed: environmental assessment and sensitivity analysis to guide future prospects. Journal of Cleaner Production, 170, 1260–1267.

[ins70066-bib-0049] van Broekhoven, S. , Doan, Q.H.T. , van Huis, A. and van Loon, J.J.A. (2014) Exposure of tenebrionid beetle larvae to mycotoxin‐contaminated diets and methods to reduce toxin levels. In Netherlands Entomological Society Meeting . FAO, Rome, Italy.

[ins70066-bib-0050] van Broekhoven, S. , Gutierrez, J.M. , De Rijk, T.C. , De Nijs, W.C.M. and van Loon, J.J.A. (2017) Degradation and excretion of the *Fusarium* toxin deoxynivalenol by an edible insect, the yellow mealworm (*Tenebrio molitor* L.). World Mycotoxin Journal, 10, 163–169.

[ins70066-bib-0051] van Huis, A. (2020) Insects as food and feed, a new emerging agricultural sector: a review. Journal of Insects As Food and Feed, 6, 27–44.

[ins70066-bib-0052] van Huis, A. (2022) Edible insects: challenges and prospects. Entomological Research, 52, 161–177.

[ins70066-bib-0053] Vidal, A. , Claeys, L. , Mengelers, M. , Vanhoorne, V. , Vervaet, C. , Huybrechts, B. *et al.* (2018) Humans significantly metabolize and excrete the mycotoxin deoxynivalenol and its modified form deoxynivalenol‐3‐glucoside within 24 hours. Scientific Reports, 8, 5255. 29588479 10.1038/s41598-018-23526-9 PMC5869592

[ins70066-bib-0054] Weiland, P. (2010) Biogas production: current state and perspectives. Applied Microbiology and Biotechnology, 85, 849–860. 19777226 10.1007/s00253-009-2246-7

[ins70066-bib-0055] Wu, F. (2004) Mycotoxin risk assessment for the purpose of setting international regulatory standards. Environmental Science & Technology, 38, 4049–4055. 15352440 10.1021/es035353n

[ins70066-bib-0056] Zeng, R.S. , Wen, Z. , Niu, G. and Berenbaum, M.R. (2013) Aflatoxin B1: toxicity, bioactivation and detoxification in the polyphagous caterpillar *Trichoplusia ni* . Insect Science, 20, 318–328. 23955884 10.1111/1744-7917.12007

