# Supplementary material for: Mycotoxins‐contaminated wheat matrices bioconversion by Tenebrio molitor larvae (Coleoptera: Tenebrionidae)
Source: Insect Sci. 2025 May 13;33(4):1691–705. doi: 10.1111/1744-7917.70066 (PMC13440987; doi:10.1111/1744-7917.70066)
Supplement: Supplementary file 1 [file INS-33-1691-s001.docx]

**Appendices**

**Table S1.** Guideline values for deoxynivalenol, zearalenone, ochratoxin A, fumonisins B1+B2 and T-2 and HT-2 toxin in feed materials and compound feed set by Commission Recommendation 2006/576/EC of 17 August 2006. Guidance value refers to a feedingstuff with a moisture content of 12 %.

| **Mycotoxin** | **Products intended for animal feed** | **Guidance value in µg/kg** |
| --- | --- | --- |
| Deoxynivalenol | Feed materials(*) |  |
|  | - Cereals and cereal products(**) with the exception of maize by-products | **8000** |
|  | - Maize by-products | **12000** |
|  | Compound feed with the exception of | **5000** |
|  | - compound feed for pigs | **900** |
|  | - compound feed for calves (< 4 months), lambs, kids and dogs | **2000** |
| Zearalenone | Feed materials(*) |  |
|  | - Cereals and cereal products(**) with the exception of maize by-products | **2000** |
|  | - Maize by-products | **3000** |
|  | Compound feed for: |  |
|  | - piglets, gilts (young sows), puppies, kittens, dogs and cats for reproduction | **100** |
|  | - adult dogs and cats other than for reproduction | **200** |
|  | - sows and fattening pigs | **250** |
|  | - calves, dairy cattle, sheep (including lamb) and goats (including kids) | **500** |
| Ochratoxin A | Feed materials(*) |  |
|  | - Cereals and cereal products(**) | **250** |
|  | Compound feed for: |  |
|  | - pigs | **50** |
|  | - poultry | **100** |
|  | - cats and dogs | **10** |
| Fumonisin B_1_+B_2_ | Feed materials(*) |  |
|  | maize and maize products(***) | **60,000** |
|  | Compound feed for: |  |
|  | - pigs, horses (*Equidae*), rabbits and pet animals | **5000** |
|  | - fish | **10,000** |
|  | - poultry, calves (< 4 months), lambs and kids | **20,000** |
|  | - adult ruminants (> 4 months) and mink | **50,000** |
| T-2 + HT-2 toxin | Compound feed for cats | **50** |

(*) Particular attention has to be paid to cereals and cereals products fed directly to the animals that their use in a daily ration should not lead to the animal being exposed to a higher level of these mycotoxins than the corresponding levels of exposure where only the complete feedingstuffs are used in a daily ration.

(**) The term ‘Cereals and cereal products’ includes not only the feed materials listed under heading 1 ‘Cereal grains and products derived thereof’ of the list of feed materials referred to in part C of the Annex to Commission Regulation (EU) No 68/2013 of 16 January 2013 on the Catalogue of feed materials (OJ L 29, 30.1.2013, p. 1) but also other feed materials derived from cereals in particular cereal forages and roughages.

(***) The term ‘Maize and maize products’ includes not only the feed materials listed under heading 1 ‘Cereal grains and products derived thereof’ of the list of feed materials referred to in part C of the Annex to Regulation (EU) No 68/2013 but also other feed materials derived from maize in particular maize forages and roughages.

**Table S2.** Effect of wheat matrix and level of mycotoxin contamination on the contamination of deoxynivalenol forms and other mycotoxins in insect exuviae^†^. Data are expressed as *µ*g/kg of exuviae. Within each single factor (wheat matrix (n=3; df=2) or the contamination level (n=3; df=2)) means followed by different capital letters are significantly different (P < 0.05), according to the Tukey test; within the values resulting from the combination of matrix level × contamination level (n=9; df=4), means followed by different lowercase letters are significantly different (P < 0.05), according to the Tukey test.

| **Mycotoxin**^‡^ | **Wheat matrix** | **Contamination level** | | | |
| --- | --- | --- | --- | --- | --- |
|  |  | **Low** | **Medium** | **High** | **Average** |
| DON | Wholegrain | 69 e | 283 c | 617 a | **323 A** |
|  | Shorts | 46 e | 201 d | 395 b | **214 B** |
|  | Bran | 58 e | 147 d | 299 c | **168 C** |
|  | **Average** | **57 C** | **210 B** | **437 A** |  |
| DON-3-G | Wholegrain | 12 e | 26 d | 55 a | **31 B** |
|  | Shorts | 11 e | 34 c | 58 a | **35 A** |
|  | Bran | 10 e | 27 d | 48 b | **28 C** |
|  | **Average** | **11 C** | **29 B** | **54 A** |  |
| DON-3-G/DON | Wholegrain | 11 b | 6 c | 6 c | **8 B** |
|  | Shorts | 16 a | 11 b | 10 bc | **12 A** |
|  | Bran | 11 b | 12 b | 10 b | **11 A** |
|  | **Average** | **12 A** | **10 B** | **9 B** |  |
| 3-ADON | Wholegrain | 5 c | 16 b | 31 a | **17** |
|  | Shorts | 5 c | 16 b | 33 a | **18** |
|  | Bran | 5 c | 15 b | 32 a | **17** |
|  | **Average** | **5 C** | **15 B** | **32 A** |  |
| DON_TOT_ | Wholegrain | 90 f | 330 c | 707 a | **376 A** |
|  | Shorts | 67 f | 256 d | 490 b | **271 B** |
|  | Bran | 79 f | 193 e | 384 c | **219 C** |
|  | **Average** | **78 C** | **260 B** | **527 A** |  |
| NIV | Wholegrain | 5 f | 23 e | 42 c | **23 C** |
|  | Shorts | 28 e | 72 b | 128 a | **76 A** |
|  | Bran | 5 f | 35 d | 68 b | **36 B** |
|  | **Average** | **13 C** | **43 B** | **79 A** |  |
| ZEA | Wholegrain | 8 e | 23 c | 44 b | **25 B** |
|  | Shorts | 8 e | 15 d | 22 c | **15 C** |
|  | Bran | 12 de | 46 b | 71 a | **43 A** |
|  | **Average** | **10 C** | **28 B** | **46 A** |  |
| ENN_TOT_ | Wholegrain | 122 ef | 127 e | 176 c | **142 C** |
|  | Shorts | 110 f | 183 c | 302 a | **199 B** |
|  | Bran | 146 d | 203 b | 295 a | **214 A** |
|  | **Average** | **126 C** | **171 B** | **257 A** |  |

^†^exuviae periodically collected during the insect rearing.

^‡^DON, deoxynivalenol; DON-3-G, deoxynivalenol-3-glucoside; DON-3-G/DON, molar ratio of deoxynivalenol-3-glucoside and deoxynivalenol; 3-ADON, 3-acetyldeoxynivalenol; DON_TOT_, total deoxynivalenol forms, sum of DON, DON-3-G and 3-ADON; NIV, nivalenol; ZEA, zearalenone; ENN_TOT_, total enniatin forms, sum of ENN A, A_1_, B and B_1_.

**Table S3.** Effect of wheat matrix and level of mycotoxin contamination on the contamination of deoxynivalenol forms and other mycotoxins in insect frass^†^. Data are expressed a *µ*g/kg of frass. Within each single factor (wheat matrix (n=3; df=2) or the contamination level (n=3; df=2)) means followed by different capital letters are significantly different (P < 0.05), according to the Tukey test; within the values resulting from the combination of matrix level × contamination level (n=9; df=4), means followed by different lowercase letters are significantly different (P < 0.05), according to the Tukey test.

| **Mycotoxin**^‡^ | **Wheat matrix** | **Contamination level** | | | |
| --- | --- | --- | --- | --- | --- |
|  |  | **Low** | **Medium** | **High** | **Average** |
| DON | Wholegrain | 15 f | 80 e | 141 d | **79 C** |
|  | Shorts | 26 f | 147 d | 266 b | **146 B** |
|  | Bran | 35 f | 184 c | 346 a | **188 A** |
|  | **Average** | **26 C** | **137 B** | **251 A** |  |
| DON-3-G | Wholegrain | 13 d | 31 c | 60 b | **35 B** |
|  | Shorts | 15 d | 35 c | 66 a | **39 A** |
|  | Bran | 14 d | 30 c | 64 ab | **36 B** |
|  | **Average** | **14 C** | **32 B** | **64 A** |  |
| DON-3-G/DON | Wholegrain | 54 a | 26 c | 28 c | **36 A** |
|  | Shorts | 37 b | 15 d | 16 d | **23 B** |
|  | Bran | 25 c | 11 d | 12 d | **16 C** |
|  | **Average** | **39 A** | **17 B** | **19 B** |  |
| 3-ADON | Wholegrain | 5 f | 13 e | 28 c | **15 C** |
|  | Shorts | 5 f | 15 d | 33 a | **18 A** |
|  | Bran | 5 f | 14 de | 31 b | **17 B** |
|  | **Average** | **5 C** | **14 B** | **31 A** |  |
| DON_TOT_ | Wholegrain | 38 e | 129 d | 235 c | **134 C** |
|  | Shorts | 51 e | 202 c | 370 b | **207 B** |
|  | Bran | 59 e | 233 c | 446 a | **246 A** |
|  | **Average** | **49 C** | **188 B** | **350 A** |  |
| NIV | Wholegrain | 13 f | 21 e | 33 d | **22 C** |
|  | Shorts | 30 d | 54 c | 107 a | **64 A** |
|  | Bran | 20 ef | 33 d | 63 b | **39 B** |
|  | **Average** | **21 C** | **36 B** | **67 A** |  |
| ZEA | Wholegrain | 0.6 f | 4 cd | 8 b | **4 B** |
|  | Shorts | 0.6 f | 2 ef | 5 c | **2 C** |
|  | Bran | 3 de | 7 b | 12 a | **7 A** |
|  | **Average** | **1 C** | **4 B** | **8 A** |  |
| ENN_TOT_ | Wholegrain | 49 ef | 54 e | 68 d | **57 C** |
|  | Shorts | 43 f | 87 c | 158 a | **96 B** |
|  | Bran | 66 d | 98 b | 154 a | **106 A** |
|  | **Average** | **53 C** | **80 B** | **127 A** |  |

^†^frass collected after the 24 h fasting period.

^‡^DON, deoxynivalenol; DON-3-G, deoxynivalenol-3-glucoside; DON-3-G/DON, molar ratio of deoxynivalenol-3-glucoside and deoxynivalenol; 3-ADON, 3-acetyldeoxynivalenol; DON_TOT_, total deoxynivalenol forms, sum of DON, DON-3-G and 3-ADON; NIV, nivalenol; ZEA, zearalenone; ENN_TOT_, total enniatin forms, sum of ENN A, A_1_, B and B_1_.

**Table S4.** Content of deoxynivalenol and total deoxynivalenol forms in insect larvae, exuviae^†^ and frass^‡^ expressed as percentage of the concentration occurred in the wheat matrix used for feeding the insects. Within each single factor (contamination level (n=3; df=2) or the DON and DON_TOT_ distribution in the different insect portions (n=3; df=2)) means followed by different capital letters are significantly different (P < 0.05), according to the Tukey test; within the values resulting from the combination of contamination level × DON and DON_TOT_ distribution in the different insect portions (n=9; df=4), means followed by different lowercase letters are significantly different, according to the Tukey test (P < 0.05).

| **Wheat matrix** | **Contamination level** | **DON (%)** | | | |  | **DON_TOT_ (%)** | | | |
| --- | --- | --- | --- | --- | --- | --- | --- | --- | --- | --- |
|  |  | **Larvae** | **Exuviae** | **Frass** | **Average** |  | **Larvae** | **Exuviae** | **Frass** | **Average** |
| Wholegrain | Low | 24 a | 11 bc | 2 d | **13 A** |  | 56 a | 12 d | 5 e | **24 A** |
|  | Medium | 13 b | 10 bc | 3 d | **8 B** |  | 25 b | 10 d | 4 e | **13 B** |
|  | High | 8 c | 11 bc | 2 d | **7 C** |  | 17 c | 11 d | 4 e | **10 C** |
|  | **Average** | **15 A** | **10 B** | **3 C** |  |  | **33 A** | **11 B** | **4 C** |  |
| Shorts | Low | 19 a | 6 cde | 3 f | **10 A** |  | 48 a | 8 d | 6 d | **20 A** |
|  | Medium | 12 b | 7 cd | 5 def | **8 B** |  | 25 b | 8 d | 6 d | **13 B** |
|  | High | 8 c | 7 cd | 5 ef | **7 C** |  | 19 c | 8 d | 6 d | **11 C** |
|  | **Average** | **13 A** | **7 B** | **5 C** |  |  | **31 A** | **8 B** | **6 B** |  |
| Bran | Low | 22 a | 9 c | 6 ef | **12 A** |  | 53 a | 10 d | 7 de | **23 A** |
|  | Medium | 12 b | 5 ef | 7 de | **8 B** |  | 25 b | 6 e | 7 de | **13 B** |
|  | High | 7 d | 5 f | 6 ef | **6 C** |  | 17 c | 6 e | 7 e | **10 C** |
|  | **Average** | **14 A** | **7 B** | **6 B** |  |  | **32 A** | **7 B** | **7 B** |  |

^†^exuviae periodically collected during the insect rearing.

^‡^frass collected after the 24 h fasting period.

DON, deoxynivalenol; DON_TOT_, total deoxynivalenol forms, sum of DON, DON-3-G and 3-ADON
